# Specialized Metabolite Profiling-Based Variations of Watercress Leaves (*Nasturtium officinale* R.Br.) from Hydroponic and Aquaponic Systems

**DOI:** 10.3390/molecules30020406

**Published:** 2025-01-19

**Authors:** Ivon Buitrago-Villanueva, Ricardo Barbosa-Cornelio, Ericsson Coy-Barrera

**Affiliations:** Bioorganic Chemistry Laboratory, Universidad Militar Nueva Granada, Cajicá 250247, Colombia; rabarbosac@gmail.com

**Keywords:** Brassicaceae, hydroponics, aquaponics, phytochemical profiling, glucosinolates

## Abstract

Watercress (*Nasturtium officinale*), a freshwater aquatic plant in the Brassicaceae family, is characterized by its high content of specialized metabolites, including flavonoids, glucosinolates, and isothiocyanates. Traditionally, commercial cultivation is conducted in submerged beds using river or spring water, often on soil or gravel substrates. However, these methods have significant environmental impacts, such as promoting eutrophication due to excessive fertilizer use and contaminating water sources with pesticides. This study aimed to explore two emerging cultivation strategies, i.e., hydroponics and aquaponics, to grow watercress and evaluate its specialized metabolite content using an untargeted metabolomic approach. The goal was to characterize metabolic profiles, identify component variations, and assess changes in metabolite accumulation at two harvest times. Two culture systems (hydroponic and aquaponic) and two harvest stages (‘baby leaf’ and traditional harvest) were examined. The results revealed 23 key metabolites, predominantly glucosinolates and flavonoids, that significantly influenced the metabolic profile discrimination, with the aquaponic system yielding the highest diversity and relative abundance of metabolites (variable importance in the projection (VIP) > 1). Important condition-related compounds were identified via cross-validation (area under the curve (AUC) > 0.7), including isorhamnetin sophoroside–glucoside and gluconasturtiin at the traditional harvest in the hydroponic system and glucoarabin at the ‘baby leaf’ stage in the aquaponic system. These findings highlight the potential of aquaponic and hydroponic systems as sustainable alternatives for watercress cultivation, offering environmental benefits and enhanced metabolite quality.

## 1. Introduction

Watercress (*Nasturtium officinale* R.Br) is a perennial freshwater aquatic plant, also known by synonyms such as *Radicula officinalis* and *Nasturtium nasturtium* [1]. It is classified as an obligate and potentially invasive hydrophyte [2,3]. The plant originates as a cultivated species from France, Germany, and England, where it is primarily consumed in salads. Presently, watercress cultivation is concentrated in the United Kingdom, the United States, and Mexico, employing floating bed systems, which also has environmental benefits, such as remediation and pollution removal [4,5]. Alternative cultivation strategies, such as hydroponics, have been explored for research purposes to evaluate primary and specialized metabolite concentrations and productivity parameters, including dry and fresh weight, leaf area, and root hair growth. These studies assess environmental factors such as temperature and photoperiod [6], nutrient loads such as sulfur and nitrogen [7], and phosphates, a primary fertilizer residue that contributes to water pollution and eutrophication [8]. Hydroponic systems, particularly vertical farming, are gaining popularity for commercial-scale cultivation due to their adaptability to non-arable soils and controlled nutrient supply [9]. Emerging techniques, such as intensive aquaponic systems, have also been applied to watercress cultivation. These systems provide sustainability benefits by eliminating effluent release into the environment and reducing the need for highly fertilized water [10].

Cultural and environmental conditions significantly influence the production/accumulation of plant-specialized metabolites [11]. Increased nitrogen fertilization in cruciferous plants enhances biomass production and the accumulation of carotenoids [7], including β-carotene, lutein, 6,5-epoxylutein, neoxanthins, and zeaxanthins, along with pigments such as chlorophylls a and b and total glucosinolates (GLS) [12]. For instance, excessive nitrogen availability leads to nitrogen accumulation in leaf tissues while maintaining GLS production, while magnesium sulfate (MgSO_4_) fertilization promotes GLS synthesis [7]. Temperature, photoperiod, and light spectrum are critical factors for gluconasturtiin production, with optimal conditions including a temperature of 15 °C, a 16:8 (light:dark) photoperiod, and a red-light spectrum [6].

Harvest time also plays a vital role in GLS production, with the highest concentrations observed 40 days post-transplant [13]. GLS concentrations are closely linked to the synthesis of their metabolic derivatives, isothiocyanates (ITC), which impart watercress with its characteristic strong, spicy flavor [14]. Notable isothiocyanates, such as sulforaphane (SFN) and phenethyl isothiocyanate (PITCE), are abundant in watercress leaves. The presence of these compounds is important since they have demonstrated the ability to inhibit cancer cell growth by modulating phase I enzymes (e.g., monooxygenases and P450s) responsible for activating carcinogens and inducing phase II enzymes that facilitate carcinogen excretion [15].

Considering that the environmental, cultural, and agronomic practices significantly affect the formation of specialized metabolites with functional and nutraceutical properties, we hypothesized that hydroponic and aquaponic systems and harvest times directly influence watercress metabolomic profiles. In this regard, optimal cultivation techniques and harvest times to maximize glucosinolate and flavonoid glycoside accumulation can be provided to benefit both producers and consumers. Additionally, this study explores chemotypic variations between hydroponic and aquaponic recirculation systems. This research is pioneering and viable, as it highlights environmentally sustainable systems for cultivating watercress with characterized metabolic profiles.

## 2. Results and Discussion

### 2.1. LC–MS-Based Phytochemical Profiling of Watercress Plants from Three Cultivation Systems

**Figure 1:**
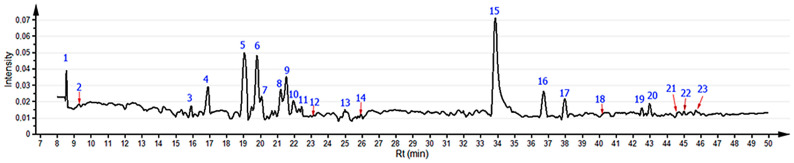
Totalized chromatogram under comparative reconstruction of the watercress samples obtained from a principal component analysis (PCA) on the entire data set (72 samples × 3937 features), using Pareto-scaled data without centering (ParN), with the Pareto variance applied as the base weight type. Blue numbers over signals represent influential metabolites detected by the employed ParN-PCA.

Phytochemical profiling and the respective correlations between metabolic profiles in watercress leaves under different cultivation conditions were determined using chemometrics on data retrieved from *m*/*z* features and the total ion chromatogram decomposition under binning, which were used to build a combined data matrix. Hence, 23 influential signals for classifying and differentiating sample groups (variable important of the projection (VIP) > 1) were recognized from a totalized chromatogram (7 min to 50 min), represented by the corresponding loadings line obtained through principal component analysis (PCA). This analysis was performed using Pareto-scaled data without centering, with the Pareto variance applied as the base weight type (Figure 1). The influential signals were distributed among watercress samples collected after propagation in hydroponic cultivation system (HCS), aquaponic cultivation system (ACS), and traditional outdoor cultivation (TOC), as well as between two harvest times such as ‘baby leaf’ (t_1_ = 21 days after transplantation, DAT) and traditional harvest (t_2_ = 42 DAT). Under this design, the analysis included 72 samples and 3937 variables (metabolite-related features).

The annotation of the statistically selected compounds is summarized in Table 1, which highlights the broad chemical diversity in watercress, with metabolites ranging from sulfur- and indole-containing compounds to phenylpropanoid derivatives. The precision of the identified compounds, as indicated by the low mass errors (below 4 ppm), validates the robustness of the analytical methods used in this study. However, three of them remained unidentified (**11**, **20**, **23**). This annotation revealed five major groups of secondary metabolites, including glucosinolates (GLS), which were categorized into branched chain (BCG), alkylthioalkyl (ATG), aliphatic (APG), and aromatic (AG) types. Among these, aliphatic GLS, such as gluconasturtiin (**16**) and glucoputranjivin (**17**), and aromatic GLS, such as glucobarbarin (**18**), were notable for their functional properties in Brassica genus plants due to their roles in plant defense and potential health benefits [16]. Branched-chain GLS, including glucobrassicanapin (**1**) and sinigrin (**3**), were also identified, with glucobrassicanapin showing a precise mass error of −2.03 ppm, reflecting high accuracy in compound annotation. Alkylthioalkyl GLS, such as glucoberteroin (**4**) and glucohirsutin (**15**), contributed to the sulfur-rich chemical diversity of the sample. In fact, compound **15** was particularly abundant in most samples.

Flavonoids, the second largest group, are synthesized via the phenylpropanoid pathway, which produces chalcone as a precursor. Enzymatic modifications of this base structure via isomerases, reductases, hydroxylases, and oxoglutarato-Fe_2_^+^ dependent dioxygenases result in a variety of flavonoids, including flavonols [17,18]. Detected flavonols included glycosylated forms such as quercetin sophoroside–glucoside (**5**), kaempferol sophoroside–glucoside (**8**), and isorhamnetin sophoroside–glucoside (**21**). These glycosylated flavonols, commonly reported in Brassica species [19], contribute to antioxidant properties and functional diversity in watercress.

Less diverse metabolites included indole-containing phytoalexins, such as 1-methoxyspirobrassinin (**6**) and spirobrassinin (**12**), both of which are synthesized in response to stress factors. These sulfur-containing metabolites, derived from the amino acid l-tryptophan, share biosynthetic pathways with GLS and play significant roles in plant detoxification processes [20,21]. Additionally, sterols, such as brassicasterol (**10**), were identified, highlighting their contribution to membrane stability and potential health benefits. Hydroxycinnamic acids (HA), such as sinapic acid (**2**) and *O*-caffeoylquinic acid (**13**), were another prominent group detected. These compounds are intermediates in lignin biosynthesis and contribute to the antioxidant capacity of watercress.

**Table 1:** Metabolite annotations of influential metabolites in *Nasturtium officinale* derived extracts after LC/MS analysis.

#	t_R_ (min)	[M+H]^+^ *m*/*z*	[M-H]^−^ *m*/*z*	Metabolite ^a^	Accurate Mass [M+H]^+^	Error (ppm) ^b^	Molecular Formula ^c^	Type ^d^
**1**	8.5	388	386	glucobrassicanapin	388.0744	−2.03	C_12_H_22_NO_9_S_2_^+^	BCG
**2**	9.4	225	223	sinapic acid	225.0768	−2.62	C_11_H_13_O_5_^+^	HA
**3**	15.9	360	358	sinigrin	360.0406	4.70	C_10_H_18_NO_9_S_2_^+^	BCG
**4**	16.9	436	434	glucoberteroin	436.0762	1.71	C_13_H_26_NO_9_S_3_^+^	ATG
**5**	19.0	789	787	quercetin sophoroside–glucoside	789.2116	−3.39	C_33_H_41_O_22_^+^	FL
**6**	19.8	281	279	1-methoxyspirobrassinin	281.0409	3.37	C_12_H_13_N_2_O_2_S_2_^+^	IPA
**7**	20.1	311	309	sinapine	311.1730	0.63	C_16_H_25_NO_5_^+^	HA
**8**	21.2	773	771	kaempferol sophoroside–glucoside	773.2159	−2.45	C_33_H_41_O_21_^+^	FL
**9**	21.5	803	801	rhamnetin sophoroside–glucoside	803.2230	1.97	C_34_H_43_O_22_^+^	FL
**10**	21.9	399	397	brassicasterol	399.3618	2.26	C_28_H_47_O^+^	E
**11**	22.4	366	364	unidentified	-	-	-	-
**12**	23.0	251	249	spirobrassinin	251.0308	2.18	C_11_H_11_N_2_OS_2_^+^	IPA
**13**	25.2	355	353	*O*-caffeoylquinic acid	355.1041	−3.30	C_16_H_19_O_9_^+^	HA
**14**	26.1	480	478	glucoibarin	480.1019	2.70	C_15_H_30_NO_10_S_3_^+^	ATG
**15**	34.0	494	492	glucohirsutin	494.1176	2.53	C_16_H_32_NO_10_S_3_^+^	ATG
**16**	36.4	424	422	gluconasturtiin	424.0727	2.03	C_15_H_22_NO_9_S_2_^+^	APG
**17**	37.9	362	360	glucoputranjivin	362.0588	−2.35	C_10_H_20_NO_9_S_2_^+^	APG
**18**	40.0	440	438	glucobarbarin	440.0677	1.91	C_15_H_22_NO_10_S_2_^+^	BCG
**19**	42.5	508	506	glucoarabin	508.1336	1.90	C_17_H_34_NO_10_S_3_^+^	ATG
**20**	43.1	310	308	unidentified	-	-	-	-
**21**	44.4	803	801	isorhamnetin sophoroside–glucoside	803.2269	−2.86	C_34_H_43_O_22_^+^	FL
**22**	45.1	454	452	glucoerysolin	454.0522	−2.44	C_12_H_24_NO_11_S_3_^+^	ATG
**23**	45.6	544	542	unidentified	-	-	-	-

^a^ Listed compounds were annotated under the combined analysis of [M+H]^+^, [M-H]^−^ quasimolecular ions accurate mass, molecular formula, and MS fragments. Numbering according to the retention time from Figure 1. The metabolite annotation was achieved at level 3 according to the confidence levels to communicate metabolite identity by high-resolution mass spectrometry (HRMS) [22]. ^b^ Error calculated between monoisotopic mass and the experimental accurate mass of the respective [M+H]^+^ ion. ^c^ Molecular formula determined from the [M+H]^+^ ion. ^d^ Classification of compounds according to chemical structures: BCG: branched chain glucosinolate; ATG: alkylthioalkyl glucosinolate; APG: aliphatic; AG: aromatic, Fl: Flavonols; IPA: indole-containing phitoalexins; HA: hidroxycinamic acids; E: esterol.

### 2.2. Pattern Recognition Through Supervised Analysis of LC–MS Data

**Figure 2:**
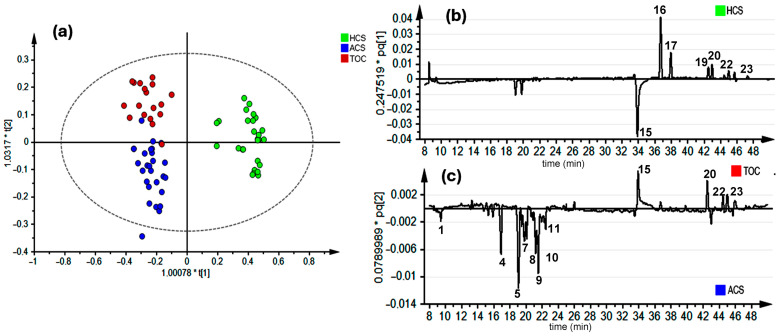
(**a**) Orthogonal partial least squares regression analysis (OPLS-DA)-derived score-plot for individual watercress leaf samples, supervised by cultivation system, i.e., hydroponic cultivation system (HCS), aquaponic cultivation system (ACS), and traditional outdoor cultivation (TOC). The data set was autoscaled using unit variance (UV); Principal Component 1 (PC1) vs. PC2 (t[1] × t[2], R^2^_Xcum_ = 0.83, Q^2^_Xcum_ = 0.72). (**b**) Loadings line obtained after combination of X loading weight p and Y loading weight q to one vector (pq[1]) obtained from PLS-DA along t[1]. (**c**) Loadings line (pq[2]) along t[2]. * represents multiplication of vector t with a constant depending on the X data distribution.

To gain deeper insights into the metabolic variations across cultivation systems, a supervised analysis using orthogonal partial least squares discriminant analysis (OPLS-DA) was performed. This analysis revealed distinct differences in the metabolite profiles of watercress leaves collected from HCS, ACS, and TOC. The scores plot (Figure 2a) demonstrated clear discrimination among the metabolite profiles of plants grown in these cultivation systems. Additionally, chemical profile reconstruction based on the loadings line obtained after a combination of X loading weight p and Y loading weight q to one vector along t[1] or t[2], using unit variance (UV) scaling, provided a clearer visualization of the contribution of individual metabolites to the observed differences. Thus, in HCS, characteristic metabolites included gluconasturtiin (**16**), glucoputranjivin (**17**), glucoarabin (**19**), and glucoerisolin (**22**) (Figure 2b), compounds primarily associated with glucosinolate biosynthesis. The ACS system exhibited higher levels of flavonoids such as quercetin (**5**), kaempferol (**8**), and rhamnetin (**9**), conjugated with the sophoroside–glucoside residue (Figure 2c). ACS also accumulated indoles like methoxyspirobrassinin (**6**) and sterols such as brassicasterol (**10**), reflecting its enhanced metabolic complexity. In contrast, TOC displayed lower metabolite diversity, with hirsutin (**15**) classified as a constitutive metabolite due to its consistently high relative abundance across all samples. These findings underscore the significant impact of cultivation systems on the metabolic profiles of watercress, with ACS demonstrating the greatest diversity and abundance of bioactive compounds, potentially linked to its sustainable nutrient cycling process [23,24].

**Figure 3:**
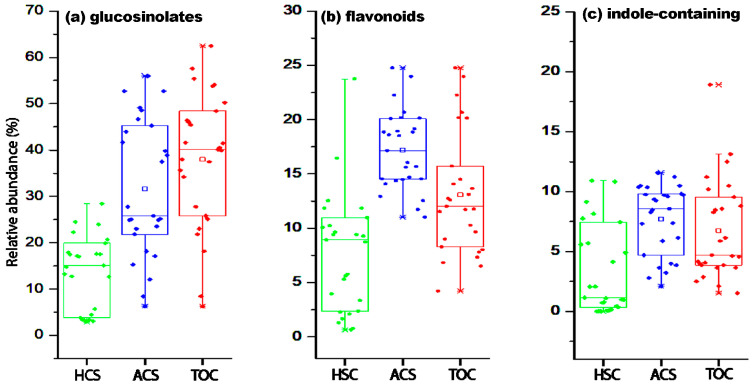
Box plots represent the distribution of the relative abundance of specialized metabolites: (**a**) glucosinolates; (**b**) flavonoids; and (**c**) indole phytoalexin.

Watercress plants propagated in HCS and ACS exhibited patterns in both leaf metabolite diversity and relative abundance. The box plots in Figure 3 illustrate the relative abundances of three specialized metabolite classes—glucosinolates (a), flavonoids (b), and indole phytoalexins (c)—in watercress leaves cultivated under HCS, ACS, and TOC. The data reveal significant variations in metabolite diversity and abundance across the three systems, providing insights into how cultivation methods influence the biosynthesis of these bioactive compounds.

The relative abundance of glucosinolates was markedly higher in ACS and TOC compared to HCS. ACS displayed the broadest range (15–65%) and the highest median, signifying its superior capacity to support glucosinolate biosynthesis. TOC also showed elevated glucosinolate levels but with slightly less variation than ACS. In contrast, HCS presented the lowest abundance, with most values below 20%. On the other hand, the flavonoid profiles followed a similar trend, with ACS and TOC exhibiting significantly higher abundances than HCS. ACS was particularly notable for its consistent flavonoid production, as evidenced by its narrow interquartile range (IQR). TOC demonstrated greater variability, with some samples showing elevated flavonoid levels. In HCS, flavonoid abundance was the lowest, rarely exceeding 10%. These findings suggest that the aquaponic and traditional systems provide conditions that enhance flavonoid synthesis, potentially due to the presence of biotic interactions and diverse nutrient sources [25,26]. Flavonoids contribute to the antioxidant properties and sensory qualities of watercress, indicating that ACS and TOC offer an advantage in producing plants with improved functional and nutritional properties. Finally, indole phytoalexins, key defense-related metabolites, also exhibited higher levels in ACS and TOC compared to HCS. ACS again emerged as the leading system, with a high median and a narrow distribution, reflecting consistent metabolite production. TOC showed slightly lower phytoalexin levels but with greater variability, possibly due to environmental fluctuations in open-field conditions. HCS displayed the lowest abundance of these metabolites, with values clustering below 5%.

These differences underscore the role of aquaponic systems in fostering the metabolic pathways involved in plant defense, likely driven by the dynamic microbial interactions and a steady supply of nitrogenous compounds in ACS. These facts are likely linked to nutrient availability and cycling, although the temperature is also a relevant factor for watercress cultivation [27]. In ACS, the continuous nutrient supply derived from fish waste decomposition, coupled with the maturation of the nitrification process, would facilitate the production of nitrogen-rich compounds such as glucosinolates [25]. In TOC, soil-based nutrient cycling may similarly promote their synthesis and perform well, although variability in metabolite production suggests less consistent nutrient availability. Conversely, the periodic replenishment of nutrient solutions in HCS appears less conducive to sustained glucosinolate production. In addition, the low phytoalexin levels in HCS may limit the plants’ ability to respond effectively to biotic stressors.

The primary difference between ACS and the other systems lies in the source and accumulation of nutrients available to the plants. In HCS and TOC, nutrient supply was provided periodically using nutrient solutions (Appendix A) every nine weeks, supplying all essential macronutrients (e.g., N, P, K, Mg, Ca) and micronutrients. In contrast, aquaponics relies on nutrient cycling from the decomposition of fish waste as the nutritional source for plants, eliminating the need for salt-based fertilization. Indeed, it is estimated that approximately 70% of the food consumed by fish is excreted in the form of ammonia and nitrate. This process typically involves a maturation phase during which nitrifying bacteria transform ammonium into nitrites and subsequently into nitrates [28]. These differences in the availability of assimilable nitrogen forms, particularly nitrates, likely explain the observed variations in metabolic profiles and relative metabolite abundance. In addition, certain nutrients—such as P, K, Cu, Fe, Mn, Zn, and S—are present in minimal or negligible amounts in fish feed, leading to potential deficiencies for plant uptake. Such nutrient limitations may influence the regulation of metabolic pathways, resulting in enhanced or reduced accumulation of specialized metabolites and ultimately causing variations in the observed metabolite profiles. These findings underscore the potential of ACS and TOC for producing watercress with superior bioactive and functional properties, with significant implications for both health and commercial value.

### 2.3. Contrasting Differential Metabolites Associated with Harvest Times in HCS and ACS

**Figure 4:**
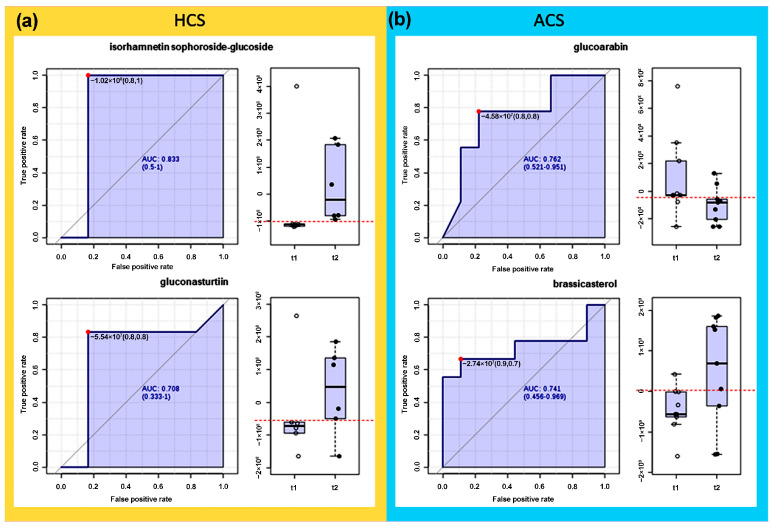
The most differential metabolites per cultivation system between harvest times t_1_ vs. t_2_ on the LC–MS-derived metabolite profile data from watercress leaves collected from (**a**) hydroponic cultivation system (HCS) and (**b**) aquaponic cultivation system (ACS). Each two-chart panel per cultivation system and differential metabolite comprises the receiver operating characteristic (ROC) curve (**left**) and the box plot showing the normalized levels per harvest time (**right**). Area under curve AUC > 0.70 as choice criteria; gluconasturtiin (AUC = 0.833), isorhamnetin sophoroside–glucoside (AUC = 0.708), glucoarabin (AUC = 0.762), and brassicasterol (10) (AUC = 0.741).

Aquaponic and hydroponic systems demonstrated the highest diversity and relative abundance of compounds. This fact prompted an additional analysis to determine those differential metabolites associated with harvest times using Receiver Operating Characteristic (ROC) curves. This analysis aimed to determine the most contrasting chemical differences regarding metabolite accumulation in the harvest stage ‘baby leaf’ (t_1_) and traditional collection (t_2_). As shown in Figure 4a, markers linked to the second harvest time t_2_ in HCS included a GLS such as gluconasturtiin (**16**) (AUC = 0.833 > 0.70) and a flavonoid like isorhamnetin sophoroside–glucoside (**21**) (AUC = 0.708 > 0.70). These findings align with previous reports indicating that gluconasturtiin content increases steadily in watercress from 10 to 40 days post-planting [13]. Moreover, vertical hydroponic systems have been shown to enhance the production of aromatic GLSs like gluconasturtiin, as well as longer-chain aliphatic GLSs [29]. Commercial hydroponic setups similar to the one used in this study also favor flavonoid production [30], which has significant implications for the organoleptic properties [31] and antioxidant activity [32] of watercress. In contrast, ACS (Figure 4b) was characterized by a broader spectrum of metabolites, including glucosinolates, flavonoids, indoles, and hydroxycinnamic acids, with the highest overall relative abundance.

Cross-validation also highlighted two influential metabolites as markers, such as the GLS glucoarabin (**19**) (AUC = 0.762 > 0.70) and the sterol brassicasterol (**10**) (AUC = 0.741). These markers were associated with the first harvest time (t_1_). Brassicasterol, a common sterol in Brassicaceae species, including rapeseed, is often accompanied by other sterols such as sitosterol, stigmasterol, and campesterol. These compounds are known for their health benefits, including reducing serum and LDL cholesterol levels and exhibiting antioxidant properties [33]. However, the discovery of **21** and **10**, **19** as differential metabolites associated with HCS and ACS, respectively, represents a novel finding in this context.

## 3. Materials and Methods

### 3.1. Design of Hydroponic and Aquaponic Cultivation Systems

The semi-intensive hydroponic and aquaponic systems were adapted from designs previously used for rocket salad cultivation [34]. Each system consisted of six planting towers connected to a 200 L tank. A 6000-L/h pump was installed inside the tank to circulate water and nutrient solution to the top of the 1.5 m towers. The solution flowed by gravity, ensuring contact with the roots of all plants before returning to the tank.

For the aquaponic system, the 200 L tank was connected to three additional tanks: a filter tank with synthetic grass to retain suspended solids, a biofilter containing plastic mesh bags with 2 cm corrugated PVC tubes to support nitrifying bacteria colonization, and a fish tank (containing 4-month *Oreochromis niloticus*) with a density of 25 individuals/m^3^ and a total capacity of 1000 L.

Each cultivation system accommodated 180 *Nasturtium officinale* plants distributed across six planting units. Each unit consisted of 30 plants (15 in the front and 15 in the back of a square tube) spaced 7.5 cm apart vertically. The test plants originated from certified seeds commercially sourced from Garden Green (Medellín, Colombia). The seeds were pre-treated by washing with distilled water and 70% ethanol, followed by germination in flowerpots filled with a commercial peat-based substrate (Pro-Mix Gtx, Impulsemillas, Tocancipá, Colombia) under controlled greenhouse conditions on the Bogotá plateau. The greenhouse environment was maintained at a temperature of 17 ± 2.8 °C, relative humidity of 65 ± 7%, altitude of 2561 m.a.s.l., total light transmission of 80 ± 8%, light diffusion of 55 ± 7%, and UV transmission (290–340 nm) of 6%. Seedlings were cultivated for 15 days until they developed two true leaves. Subsequently, 180 seedlings were transplanted into each of the experimental cultivation systems, i.e., ACS and HCS, both maintained under the same greenhouse conditions.

### 3.2. Metabolite Extraction

Watercress leaves (20 g) were harvested 21 days after transplant (DAT) (‘baby leaf’), and the traditional harvest took place at 42 DAT. Collected leaves were quenched by freezing in liquid nitrogen and subsequently lyophilized. The dried material was extracted with cold 95% ethanol in a 1:2 (*w*/*v*) sample-to-solvent ratio and carefully stirred with a temperate orbital shaker (4 °C) for 30 min. The mixture was filtered and collected; this process was repeated two additional times. The combined filtrates were concentrated using a rotary evaporator (IKA^®^ RV10) at 35 °C, 90 rpm, and 110 psi to obtain the raw extract.

### 3.3. Obtaining Chromatographic Profiles by Liquid Chromatography Coupled to Mass Spectrometry (HPLC–MS)

The crude extract was filtered through a 0.22-µm silicone/PTFE membrane (MS^®^PTFE syringe filter, Membrane Solutions) and dissolved in HPLC-grade absolute ethanol (Emsure^®^, Merck, Darmstadt, Germany) at a final concentration of 2.5 mg/mL. Chromatographic analysis was initially performed using a Prominence HPLC system (Shimadzu, Columbia, MA, USA) coupled with a diode array detector (DAD) SPD-M20A and a mass spectrometry detector (MSD) LCMS-2020 equipped with an electrospray ionization (ESI) interface and a single quadrupole analyzer. Component separation was carried out using a Premier C18 column (4.6 mm × 150 mm, 3.5 µm) and a binary mobile phase consisting of 0.005% formic acid (eluent A) and acetonitrile (eluent B). The gradient started at 5% B, gradually increased to 95% B over 55 min, held for 5 min, then decreased to 5% B and held until 65 min. The flow rate was 0.5 mL/min, with an injection volume of 20 µL. Detection was monitored at 270 nm. The ESI was operated in positive and negative ion modes (scan range 100–1500 *m*/*z*), with a desolvation line temperature of 250 °C, a nitrogen nebulizer gas flow of 1.5 L/min, a drying gas flow of 15 L/min, and a detector voltage of 1.4 kV. Additionally, LC-HRMS analysis was conducted using an Agilent Technologies 1260 Liquid Chromatography system coupled to a quadrupole-time-of-flight (Q-ToF) mass analyzer with dual Agilent jet stream electrospray ionization (AJS ESI). Chromatographic parameters were identical to those above. The AJS ESI source parameters included a capillary voltage of 3500 V, a drying gas flow of 8 L/min, a gas temperature of 325 °C, a nebulizer pressure of 50 psi, a sheath gas temperature of 350 °C, and a sheath gas flow of 11 L/min. The Q-ToF parameters involved a fragmentor voltage of 175 V, a skimmer voltage of 65 V, and an octapole radiofrequency peak-to-peak voltage (OCT RF Vpp) of 750 V.

### 3.4. Data Collection and Multivariate Analysis

**Figure 5:**
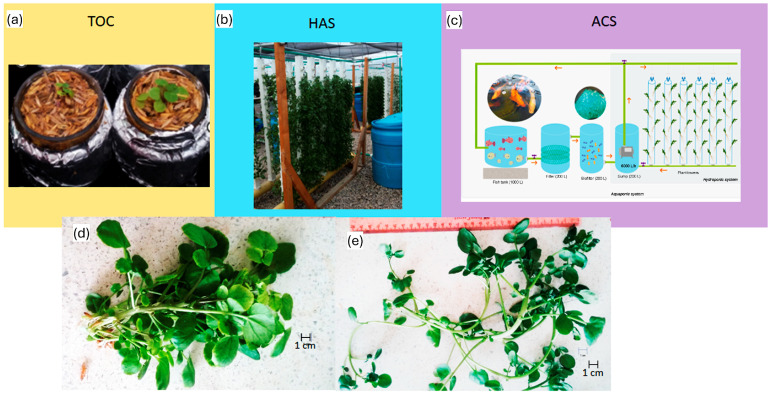
(**a**) Traditional outdoor culture (TOC); (**b**) hydroponic cultivation system (HAS); (**c**) aquaponic cultivation system (modified from a previous design [34]; (**d**) first harvest time (t_1_) or ‘baby leaf’; and (**e**) second harvest time (t_2_) or traditional harvest.

Plants were harvested from three cultivation systems: hydroponic (HCS); aquaponic (ACS); and traditional outdoor culture (TOC) with rice husk substrate and nutrient solution (Figure 5a–c). Two harvest times were evaluated: ‘baby leaf’ (21 DAT) (Figure 5d) and traditional harvest (42 DAT) (Figure 5e). Each plant served as an experimental unit with three replicates (*n* = 72).

Mature leaves were collected (excluding those plants at the edges), yielding 20 g of fresh material per sample. Each sample was extracted and analyzed by LC–MS (vide supra). The LC–MS-derived data were processed to extract *m*/*z* features (variables) for each plant sample (observations) and used to generate a feature intensity table (FIT) (variables × observations). This table was created using MZmine 2.0, applying previously established preprocessing parameters, including peak alignment, normalization by sum, and autoscaling [34]. In addition, the total ion chromatograms (TIC) were sectioned every 0.2 s (binning) and converted into ASCII format. The resulting dataset was normalized and autoscaled in MS Excel^®^ 2013 and aligned using MATLAB R2013a software. The processed data were analyzed in SIMCA 14.0 (Umetrics Inc., Rochester, NY, USA) using unsupervised principal component analysis (PCA) and hierarchical cluster analysis (HCA). Based on PCA and HCA insights, supervised orthogonal partial least squares regression analysis (OPLS-DA) was performed to identify differential metabolites associated with cultivation conditions and sample classifications. These analyses employed Pareto-scaled data without centering and UV-scaled with centering, with the Pareto and unit variance applied as the base weight types. Chemical profiles were statistically reconstructed based on the loading lines obtained after a combination of X loading weight p and Y loading weight q to one vector along t[1] or t[2].

### 3.5. Annotation of Statistically Selected Metabolites

Metabolites showing statistically significant enhancements (VIP > 1) by culture systems and harvest times were annotated based on accurate mass data obtained from high-resolution mass spectrometry (HRMS). The molecular formula for each selected feature was deduced from the precise mass measurements of the quasimolecular ion [M-H]^−^ using the ChemCalc online tool, ensuring a mass error of ≤5.0 ppm. The annotation process involved a comprehensive analysis of HRMS data, including accurate mass, molecular formula, and MS fragmentation patterns, employing the confidence levels to communicate metabolite identity by HRMS [22]. This was further complemented by phylogenetic filtering, chromatographic behavior analysis (when available), and comparisons with the established literature and databases such as the Dictionary of Natural Products, KNApSAcK, and PubChem. This multifaceted approach ensured reliable annotation by aligning HRMS-derived insights with biochemical, chromatographic, and phylogenetic information.

### 3.6. Top-Ranked Metabolites by Harvest Time Effect in Each Culture System

Validation of top-ranked metabolites associated with harvest times was conducted using area-under-the-curve (AUC) data from retention time intensities. The data were transformed into CSV format and analyzed using MetaboAnalyst V5.0. Data normalization and mean centering were applied to construct receiver operating characteristic (ROC) curves via Monte Carlo cross-validation. Metabolites with AUC values > 0.70 were selected as markers, meeting the minimum threshold for the selection [35,36].

## 4. Conclusions

The clean production schemes evaluated (hydroponics and aquaponics) proved suitable for cultivating watercress (*N. officinale*) under greenhouse conditions. Both systems facilitated the production of watercress leaves with a high content of metabolites known for their functional and nutraceutical properties. However, the aquaponic system (ACS) demonstrated a clear advantage, yielding metabolic profiles with the highest diversity and relative abundance of compounds. Thus, the findings highlight significant differences in metabolite diversity and relative abundance across the cultivation systems, with ACS and HCS demonstrating superior metabolite diversity compared to TOC. Aquaponics (ACS) exhibited a notable enrichment of glucosinolates, flavonoids, and indole derivatives, suggesting that nutrient sources derived from fish waste decomposition and nitrifying bacterial activity may enhance the bioavailability of key nitrogenous compounds, driving the production of specialized metabolites. This outcome is likely attributed to differences in nutrient availability; while hydroponic systems (HSC) rely on periodic nutrient supplementation, ACS benefits from continuous nutrient cycling derived from fish waste. This nutrient source, rich in nitrates, aligns with the watercress’s capacity to accumulate nitrates, though excess fertilization does not always translate to enhanced phytochemical biosynthesis. Conversely, HCS showed a specific enhancement in aromatic glucosinolates, such as gluconasturtiin, which aligns with previous reports linking hydroponic systems to increased aromatic metabolite synthesis. The ROC curve analysis identified markers associated with harvest times, emphasizing that gluconasturtiin and isorhamnetin sophoroside–glucoside were strongly linked to the second harvest in HCS, while glucosinolates such as glucoarabin and sterols like brassicasterol were characteristic of ACS. These markers provide valuable insights into optimal harvest times for maximizing the production of health-beneficial metabolites. The experimental design, which included the transplantation of seedlings into standardized cultivation systems and the use of LC–MS-derived metabolomic data, enabled robust statistical analyses. Tools such as OPLS-DA effectively highlighted the influence of cultivation systems on metabolite profiles, reinforcing the importance of system-specific conditions in determining plant biochemical composition. Ultimately, the results underscore the potential of aquaponics and hydroponics as sustainable cultivation systems that enhance watercress phytochemical profiles. These findings hold significant implications for agricultural practices and functional food development, particularly in optimizing systems for producing crops with enriched bioactive compounds for health-promoting properties. Further research should explore the scalability of these systems and their long-term impacts on metabolite production.

## Data Availability

The datasets generated during and/or analyzed during the current study are available from the authors upon reasonable request.

## Abbreviations

The following abbreviations are used in this manuscript:

VIP	variable important of the projection
PCA	Principal component analysis
HCS	Hydroponic cultivation system
ACS	Aquaponic cultivation system
TOC	Traditional outdoor cultivation
DAT	Days after transplantation,
OPLS-DA	Orthogonal partial least squares discriminant analysis
LC–MS	Liquid chromatography coupled with mass spectrometry
GLS	Glucosinolates

## Appendix A

**Table A1:** Concentrated nutrient solution. Modified from Guardabaxo et al. 2020, [37].

**Compounds in Stock A**	**Formula**	**Concentration (g/L) ^a^**
Potassium nitrate	KNO_3_	183
Monoammonium phosphate	(NH_4_)H_2_PO_4_	117
Calcium nitrate	Ca(NO_3_)_2_	60
**Compounds in stock B**		
Magnesium sulphate	MgSO_4_	73
Iron chelate	11.3% Fe	6
**Compounds in stock C**		
Manganese sulphate	MnSO_4_H_2_O	5
Boric acid	H_3_BO_3_	3
Zinc sulfate	ZnSO_4_	1.7
Copper sulphate	CuSO_4_	1
Ammonium molybdate	(NH_4_)_6_Mo_7_O_24_	0.2

^a^ The preparation of the hydroponic solution involved the corresponding aliquot at a final concentration of 5 mL/L of stock A, 0.4 mL/L of stock B, and 1.6 mL/L of micronutrients.
